# Preliminary hematological values and blood cell morphology of wild-caught Spotted scat (Scatophagus argus) from Southern Thailand

**DOI:** 10.14202/vetworld.2026.3742-3754

**Published:** 2026-08-27

**Authors:** Kanpapat Boonchuay, Kannawee Swangneat, Pachara Khiewthong, Thitapron Mangkang, Tran Nhat Thang, Nikom Srikacha, Pornchai Pornpanom

**Affiliations:** 1 Akkhraratchakumari Veterinary College, Walailak University, Nakhon Si Thammarat 80160, Thailand.; 2 One Health Research Center, Walailak University, Nakhon Si Thammarat 80160, Thailand.; 3 Faculty of Animal Science and Veterinary Medicine, Thai Nguyen University of Agriculture and Forestry, Thai Nguyen 24119, Vietnam.; 4 Department of Animal Science, Faculty of Natural Resources, Rajamangala University of Technology Isan, Sakon Nakhon 47160, Thailand.; 5 Informatics Innovation Center of Excellence, Walailak University, Nakhon Si Thammarat 80160, Thailand.

**Keywords:** aquaculture, bioindicator, complete blood count, euryhaline fish, hematology, One Health, Scatophagus argus, Southern Thailand

## Abstract

**Background and Aim:**

Spotted scat (*Scatophagus argus*) is a euryhaline teleost of increasing importance in aquaculture, ornamental fisheries, and environmental monitoring across the Indo-Pacific region. Hematological evaluation is a valuable tool for assessing fish health, physiological status, and environmental quality; however, species-specific reference data for spotted scat remain scarce. This study aimed to characterize blood cell morphology and morphometry and establish preliminary hematological reference values for wild-caught spotted scat from Southern Thailand to support veterinary diagnostics, future research, and biomonitoring applications.

**Materials and Methods:**

Twenty-six wild-caught spotted scats were collected from Tha Sung Canal, Nakhon Si Thammarat Province, Thailand, between June and August 2025. Fish were anesthetized with clove oil before blood collection from the caudal vein into ethylenediaminetetraacetic acid tubes. Wright-stained blood smears were prepared for microscopic evaluation of blood cell morphology and morphometry. Hemoglobin (Hb) concentration was measured using an automated hematology analyzer, whereas packed cell volume (PCV), erythrocyte and leukocyte counts, differential leukocyte counts, thrombocyte counts, and erythrocyte indices were determined using standard manual hematological methods. Descriptive statistics were generated, and leukocyte morphometry was compared using the Kruskal-Wallis test followed by Dunn’s multiple-comparison test.

**Results:**

Four leukocyte types were identified: neutrophils, eosinophils, monocytes, and lymphocytes. Neutrophils and monocytes were the largest leukocytes and exhibited similar diameters (p > 0.05), although monocytes possessed a characteristically deeper blue cytoplasm that facilitated differentiation. Eosinophils occurred as small and large forms, whereas lymphocytes represented the predominant circulating leukocyte population. Juvenile male fish had a mean PCV of 0.24 ± 0.07 L/L, Hb concentration of 74.41 ± 20.94 g/L, erythrocyte count of 1.80 ± 0.68 × 10¹²/L, and leukocyte count of 32.47 ± 14.81 × 10⁹/L. Lymphocytes (16.14 ± 7.27 × 10⁹/L) were the predominant leukocyte subtype, followed by monocytes, neutrophils, and eosinophils.

**Conclusion:**

This study provides the first comprehensive description of blood cell morphology together with preliminary hematological reference values for wild-caught spotted scat from Southern Thailand. These baseline data enhance diagnostic capacity for piscine hematology, support health assessment of ornamental and cultured fish, and provide a valuable foundation for future studies investigating environmental stress, disease, and the use of spotted scat as a bioindicator of freshwater, brackish, and mangrove ecosystems within the One Health framework.

## INTRODUCTION

Spotted scat (*Scatophagus argus* [Linnaeus, 1766]), also known as spotted spadefish, spotted butterfish, tiger scat, or argus fish, is a teleost species belonging to the family Scatophagidae and the order Perciformes [1, 2]. The species is widely distributed throughout nearshore environments of the Indo-Pacific region, including India, Sri Lanka, Thailand, Malaysia, Vietnam, the Philippines, China, and Australia [1–3]. It is an economically important food fish in Southeast Asia [2, 4], providing a valuable source of protein and essential amino acids [5, 6], and is also highly valued in the ornamental fish trade. In Thailand, *S. argus* has become an increasingly important aquaculture species because it can be successfully cultured in floating cage systems, particularly in Songkhla Lagoon [7].

*S. argus* exhibits marked euryhalinity and inhabits freshwater, brackish, and marine ecosystems [8]. Larvae predominantly occur in freshwater and brackish environments, whereas adults are mainly distributed in marine habitats [3]. The species is also commonly found in natural and restored mangrove forests, as well as in abandoned shrimp farms. Owing to its broad ecological distribution, *S. argus* likely plays important roles in maintaining the structure and function of aquatic ecosystems through predation and nutrient cycling, as reported for other fish species [9, 10]. Furthermore, its occurrence across diverse aquatic environments highlights its potential as a bioindicator of environmental quality because hematological parameters are sensitive indicators of physiological responses to aquatic pollution [11].

Although hematological evaluation is not yet routinely incorporated into aquaculture management or fisheries research [12], complete blood counts (CBC) are valuable tools for assessing fish health, physiological status, and welfare [13]. Their importance has increased further due to growing concerns about environmental degradation and aquatic pollution [14, 15]. Routine CBC analysis in fish is generally performed manually because nucleated blood cells limit the accuracy of conventional automated hematology analyzers [16]. This limitation is particularly evident during white blood cell (WBC) enumeration because incomplete lysis of nucleated erythrocytes (RBCs) and thrombocytes can interfere with automated cell counting [17].

Automated hematology analyzers primarily use either electrical impedance or optical detection technologies [18]. The impedance method detects changes in electrical resistance as cells pass through an aperture, enabling cell enumeration and volume estimation based on cell size [19]. In contrast, optical systems classify cells by analyzing light scattering generated by differences in cellular refractive indices [19]. However, in vertebrates with nucleated erythrocytes, naked erythrocyte nuclei, thrombocytes, and lymphocytes share similar physical characteristics, making accurate differentiation difficult with either impedance- or light-scatter-based technologies [20]. Consequently, manual hematological assessment remains the preferred method for most teleost species.

Species-specific interpretation of CBC results requires reliable hematological reference intervals (RIs), which are influenced by numerous biological and environmental factors, including species, age, sex, nutritional status, water temperature, and water quality [21–25]. Furthermore, automated RBC and WBC counts require validation against manual methods to ensure analytical accuracy because hematological findings may be affected by stress, sample handling, laboratory methods, and the accuracy of blood cell identification [16, 26–29]. Despite the ecological and economic importance of *S. argus*, comprehensive information describing the morphology, morphometry, and hematological characteristics of peripheral blood cells in wild-caught individuals remains unavailable.

Previous studies on *S. argus* have primarily addressed its ecology, nutrition, aquaculture potential, reproductive biology, and genetic diversity. However, comprehensive hematological characterization of wild populations remains limited. In particular, detailed descriptions of blood cell morphology and morphometry have not been integrated with preliminary quantitative hematological values for this species. This deficiency limits the interpretation of CBC findings in clinical, aquaculture, conservation, and environmental monitoring contexts. In addition, the absence of species-specific baseline data limits the use of *S. argus* as a bioindicator for assessing environmental health across freshwater, brackish, marine, and mangrove ecosystems. Establishing such baseline information is therefore essential for improving veterinary diagnostic capacity, facilitating comparisons among populations and habitats, and supporting future investigations of physiological responses to disease, husbandry conditions, and environmental stressors.

This study aimed to characterize the morphology and morphometry of peripheral blood cells and establish preliminary hematological values for wild-caught *S. argus* from Southern Thailand. The findings are expected to provide baseline information for routine piscine hematology, improve diagnostic and clinical services for ornamental and cultured fish, and support future investigations of fish health and environmental stress. The resulting data may also strengthen the use of *S. argus* as a potential bioindicator of freshwater, brackish, marine, and mangrove ecosystems and contribute to aquatic conservation and sustainable resource management in accordance with Sustainable Development Goal 14 (Life Below Water).

## MATERIALS AND METHODS

### Ethical approval

All experimental procedures involving animals were reviewed and approved by the Walailak University Institutional Animal Care and Use Committee (Approval No. WU-ACUC-68002). The study was conducted in accordance with the institutional guidelines for the care and use of experimental animals and internationally accepted principles for the ethical use of animals in research. During the anesthetic procedure, all *S. argus* individuals were continuously monitored for loss of equilibrium, cessation of voluntary swimming, and absence of responses to external stimuli to ensure an appropriate anesthetic depth before blood collection. Blood sampling was performed under humane conditions to minimize stress, pain, and prolonged suffering. Following sample collection, the fish were transferred to aerated recovery tanks and monitored until normal swimming behavior and equilibrium were restored, after which they were released back into their natural habitat.

### Study period and location

The study was conducted between June and August 2025. Wild *S. argus* were collected from Tha Sung Canal, Tha Sala District, Nakhon Si Thammarat Province, Thailand (8°39′ N, 99°56′ E).

### Study design

This was a descriptive cross-sectional study designed to characterize peripheral blood cell morphology, morphometry, and preliminary hematological values in wild-caught *S. argus*. Blood samples collected from apparently healthy fish were subjected to microscopic examination and CBC analysis using standardized hematological procedures.

### Fish and sampling sites

A total of 26 wild *S. argus*, weighing 18.10-114.80 g, were captured using a cast net from Tha Sung Canal. Information regarding the natural diet of the sampled fish was unavailable. However, a previous study conducted in a nearby province reported that the diet of *S. argus* consists primarily of crustaceans, polychaetes, mollusks, algae, and fish eggs [30]. Water quality parameters, including temperature, pH, dissolved oxygen, ammonia, and nitrite concentrations, were not measured during the sampling period.

Sex was determined according to external head morphology as described by Barry and Fast [8]. Males were identified by the presence of a slightly indented curve above the eye, whereas females exhibited a smooth and continuously sloping head profile. Based on these criteria, 25 fish were identified as males and one as a female. Age classification followed previously established body weight criteria, in which adult males weigh ≥80 g and adult females weigh ≥150 g [8]. Accordingly, one male was classified as an adult, whereas the remaining 25 fish (24 males and one female) were classified as juveniles.

### Fish anesthesia and blood sample collection

Fish were anesthetized using a 30 ppm clove oil solution prepared by diluting 6 mL of a 10% clove oil stock solution in 20 L of aerated water until loss of equilibrium and cessation of opercular movement were observed (3-5 min) [31]. Approximately 0.5 mL of blood was collected from the caudal vein using a 24-gauge needle attached to a 1 mL syringe.

Immediately after collection, blood samples were transferred into tubes containing ethylenediaminetetra-acetic acid (EDTA) (Quetainer™, Cangzhou Fukang Medical Supplies, Hebei, China). EDTA was selected because it is the anticoagulant most commonly used in fish hematology. Nevertheless, previous studies have demonstrated that EDTA may induce erythrocyte swelling in some teleost species [32], whereas heparin may better preserve blood cell morphology in certain fish species.

### Microscopic examination of blood cells

Three blood smears were prepared from each fish using the push-slide technique on clean glass slides. Air-dried smears were stained with Wright's stain (Wright eosin methylene blue solution; Merck KGaA, Darmstadt, Germany) according to previously described procedures [31]. Smears were immersed in the stain for 6 min, which simultaneously served as the fixation step [33], thereby eliminating the need for methanol fixation. Subsequently, buffer solution was added for 8 min before the slides were gently rinsed with running tap water.

Blood smears were initially screened under 400× magnification across 100 microscopic fields to identify blood cell abnormalities, hemoparasites, or staining artifacts. Detailed cellular evaluation was subsequently performed at 1,000× magnification. Digital images were captured using an Olympus BX43 microscope equipped with a DP27 digital camera and CellSens software (version 1.18; Olympus Corporation, Tokyo, Japan). Subsequent references to CellSens software refer only to Olympus.

Morphometric analysis was performed using 100 randomly selected cells of each blood cell type from the monolayer region of each smear. Basophils were not observed in any specimen. Erythrocyte length, width, and surface area, together with leukocyte diameter, were measured using CellSens software. All microscopic examinations were independently performed by two trained veterinary technicians who were blinded to the sample identity.

### CBC analysis in *S. argus*

Hemoglobin (Hb) concentration was measured using an URIT-300Plus™ hematology analyzer (URIT Medical Electronic Co., Ltd., Guilin, China) with EDTA-anticoagulated whole blood. All remaining hematological parameters were determined manually by trained veterinary technicians in accordance with established hematological procedures [34–36].

Packed cell volume (PCV) was determined using the microhematocrit method following centrifugation at 14,000 × *g* for 5 min. Total RBC and WBC counts were determined manually after dilution (1:200) using in-house-prepared Natt and Herrick's solution [37]. Cell counts were performed using a Neubauer hemocytometer under 400× magnification, and all counts were independently verified by two trained veterinary technicians.

RBC indices were calculated using the following equations:

MCV (fL) = PCV (%) × 10 / RBC (×10⁶/µL)

MCH (pg) = Hb (g/dL) × 10 / RBC (×10⁶/µL)

MCHC (g/dL) = Hb (g/dL) × 100 / PCV (%)

Differential WBC counts were performed by examining 100 leukocytes per blood smear under 1,000× magnification. Thrombocytes were enumerated per 100 WBCs. Relative leukocyte percentages were converted to absolute leukocyte counts using the following equation:

Absolute leukocyte count (×10⁹/L) = Relative leukocyte percentage × Total WBC count (×10⁹/L) / 100

### Statistical analysis

All statistical analyses were performed using R software (version 4.5.3) [38]. Preliminary hematological values were summarized using descriptive statistics, including the mean, median, standard deviation (SD), minimum, and maximum values. Data distributions were assessed visually using histograms generated with the ggplot2 package. Potential outliers were examined using boxplots generated with the ggplot2 package and were retained unless considered biologically implausible.

Differences in leukocyte diameter among WBC types were evaluated using the Kruskal-Wallis test followed by Dunn's multiple-comparison test. Statistical significance was established at p < 0.05.

## RESULTS

### Blood cell morphology and morphometry

All 26 wild-caught *S. argus*, comprising 24 juvenile males, one adult male, and one juvenile female, showed no clinical signs or external lesions. Microscopic examination of Wright-stained blood smears revealed erythrocytes (RBCs), leukocytes (WBCs), and thrombocytes.

**Figure 1:**
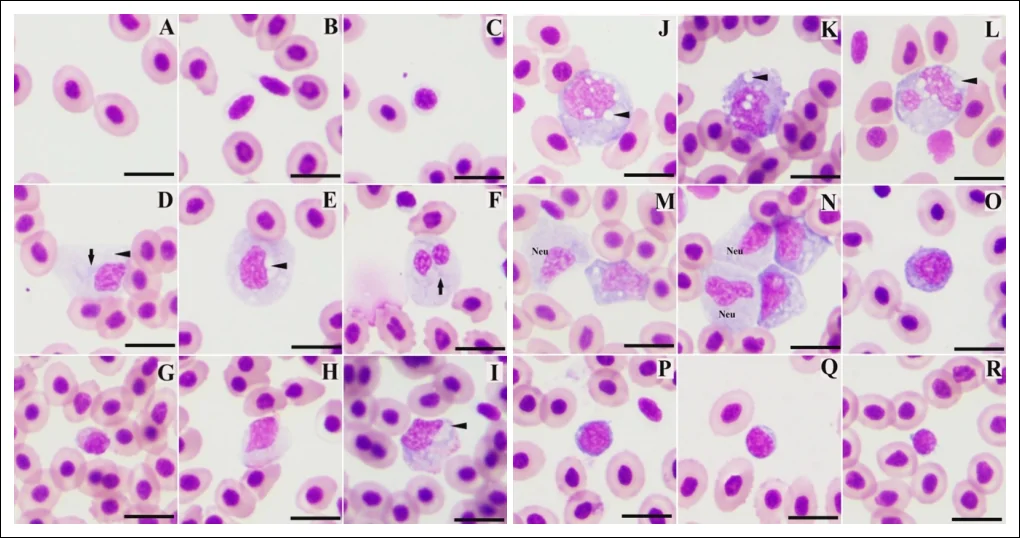
Blood cells of wild-caught *Scatophagus argus*. Mature erythrocytes are oval with an oval nucleus and acidophilic cytoplasm (A). Thrombocytes are oval with an oval nucleus and colorless cytoplasm (B), whereas occasional round thrombocytes are present (C). Neutrophils have round-to-oval nuclei (D, E), with occasional bilobed nuclei (F). Small eosinophils (G) and moderate-to-large eosinophils (H, I) are observed. Monocytes have round-to-oval nuclei (J, K, M–O), whereas some exhibit irregular nuclei (L). Lymphocytes are shown in panels P–R. Vacuoles (arrowheads) are observed in monocytes and occasionally in neutrophils, eosinophils, and lymphocytes. A dark basophilic zone (arrow) is present in some neutrophils. Eosinophil granules are brightly eosinophilic with indistinct outlines, with occasional small rod-shaped structures (I). Neu = Neutrophil. Scale bar = 10 μm.

RBCs were oval, with a centrally positioned oval nucleus (Figure 1A). Their median length and width were 7.92 µm (IQR = 0.56) and 5.70 µm (IQR = 0.49), respectively (Table 1). Thrombocytes occurred in two distinct morphological forms: oval and round. Oval thrombocytes were predominant (Figure 1B) and had a median length of 7.31 µm (IQR = 1.31) (Table 1). Round thrombocytes were less frequently observed (Figure 1C) and had a median diameter of 3.79 µm (IQR = 0.62).

**Four WBC types were identified:** neutrophils, eosinophils, monocytes, and lymphocytes. Neutrophils were round cells with abundant cytoplasm that ranged from colorless or pale gray (Figure 1D) to gray (Figures 1E and F). A localized zone of dark basophilia was occasionally observed (Figures 1D and E), and cytoplasmic vacuoles were also present in some cells (Figure 1D and E). The nucleus was generally round-to-oval (Figure 1D and E), although irregular and bilobed forms were occasionally detected (Figure 1F).

Eosinophils were round, with an eccentrically positioned round-to-oval nucleus. Their cytoplasm was generally colorless (Figures 1G and H) but occasionally appeared gray-blue and contained vacuoles (Figure 1I). Bright eosinophilic granules with indistinct margins were present in the cytoplasm and sometimes appeared as small rod-shaped structures (Figure 1I). Eosinophils were classified into small and large forms (Table 1). Small eosinophils predominated and had a median diameter of 5.29 µm (IQR = 1.18), whereas large eosinophils were observed only occasionally and had a median diameter of 7.77 µm (IQR = 1.55).

Monocytes were round cells with abundant, deeply basophilic cytoplasm. Their nuclei were pleomorphic, most commonly round-to-oval (Figures 1J and K), but occasionally irregular (Figures 1M and N). Monocyte morphology closely resembled that of neutrophils; however, monocytes were distinguishable by their more deeply blue cytoplasm. Small monocytes resembling lymphocytes were also observed (Figure 1O). The median monocyte diameter was 9.96 µm (IQR = 1.59), which was slightly smaller than the median neutrophil diameter of 10.30 µm (IQR = 1.46); however, the difference was not statistically significant (p > 0.05).

Lymphocytes were small, round cells with an eccentrically positioned round nucleus and a limited amount of blue cytoplasm (Figures 1P and Q). Short cytoplasmic processes were occasionally observed (Figure 1R). Their median diameter was 5.52 µm (IQR = 1.33). Lymphocytes were sometimes difficult to distinguish from round thrombocytes due to their similar size; however, cytoplasmic staining aided differentiation, as round thrombocytes had colorless cytoplasm.

**Table 1:** Morphometry of blood cells in wild-caught Scatophagus argus from Nakhon Si Thammarat, Southern Thailand.

**Blood cells**	**Unit**	Median (IQR)*	**Min–Max**
Erythrocytes (n = 100)			
Length	μm	7.92 (0.56)	6.60–8.92
Width	μm	5.70 (0.49)	4.85–6.61
Area	μm²	38.10 (4.73)	30.00–48.84
Diameter of leukocytes			
Neutrophils (n = 100)	μm	10.30 (1.46)ᵃ	8.41–12.40
Large eosinophils (n = 25)	μm	7.77 (1.55)ᵇ	7.06–9.99
Small eosinophils (n = 75)	μm	5.29 (1.18)ᶜ	3.92–7.20
Monocytes (n = 100)	μm	9.96 (1.59)ᵃ	7.68–14.14
Lymphocytes (n = 100)	μm	5.52 (1.33)ᶜ	3.77–8.47
Round thrombocytes			
Diameter (n = 25)	μm	3.79 (0.62)	3.05–5.27
Oval thrombocytes			
Length (n = 75)	μm	7.31 (1.31)	5.35–8.96
Width (n = 75)	μm	3.58 (0.74)	2.34–4.56

*Data are presented as median with IQR in brackets. Different superscript letters (ᵃ–ᶜ) for leukocyte diameter indicate statistically significant differences among cell types (p < 0.05). IQR = Interquartile range.

### Preliminary hematological values

**Figure 2:**
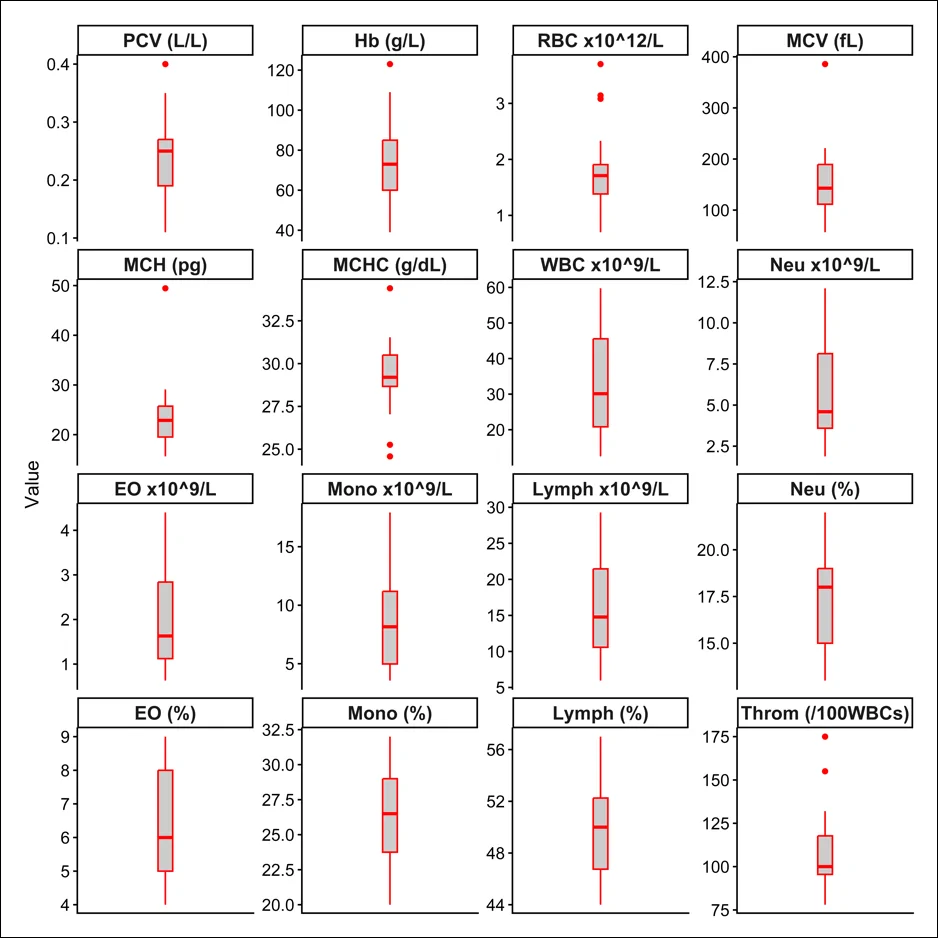
Box plots showing the median values and interquartile range of hematological variables in juvenile male *Scatophagus argus*. Outliers are displayed as individual data points.

Hematological measurements from the 24 juvenile male *S. argus* were visually examined for potential outliers (Figure 2). Outliers were identified for PCV, Hb concentration, total RBC count, MCV, MCH, MCHC, and thrombocyte count per 100 WBCs. Because all fish were collected from the same environment and were likely exposed to similar dietary conditions, these extreme values were retained and were considered potentially related to biological variability and the limited sample size.

**Figure 3:**
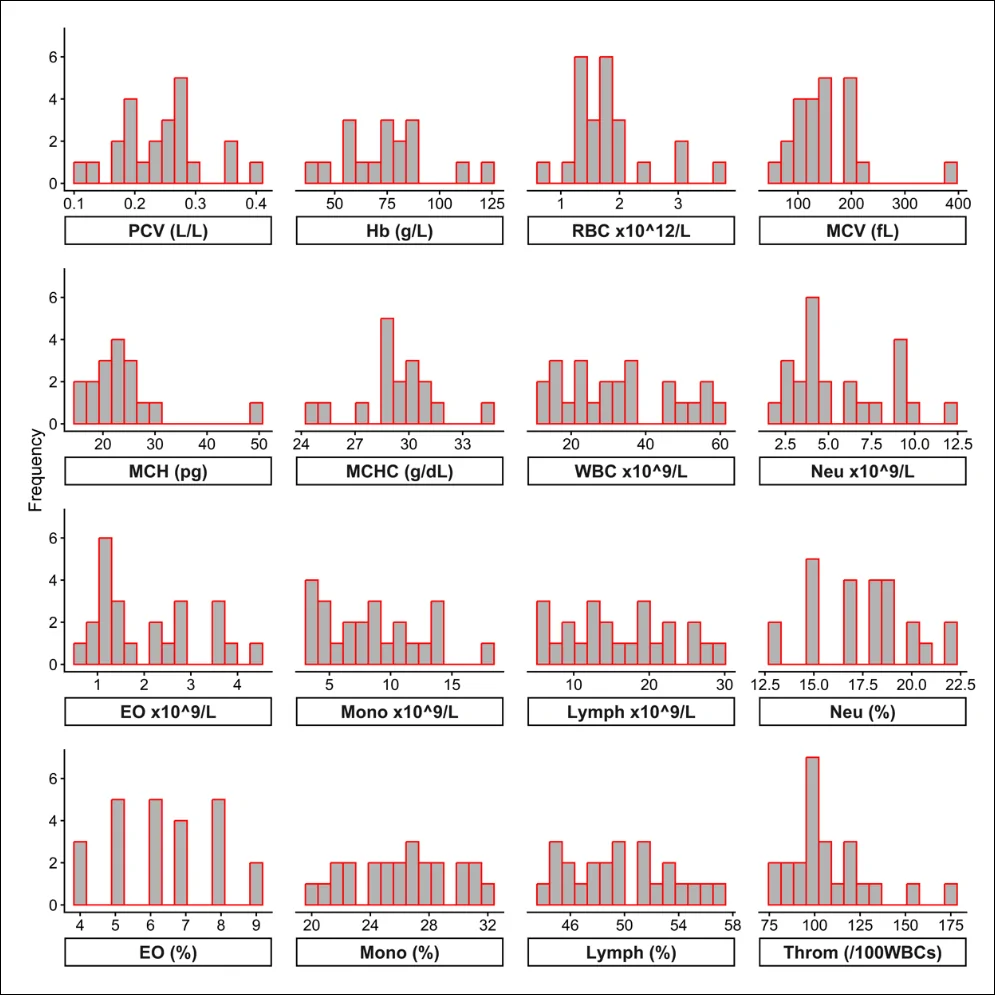
Histograms showing the distribution of hematological variables in juvenile male *Scatophagus argus*. The x-axis represents the measured values, whereas the y-axis represents the frequency of occurrence.

The preliminary hematological values are summarized in Table 2, and their distributions are presented as histograms in Figure 3. Juvenile males had a mean PCV of 0.24 ± 0.07 L/L, Hb concentration of 74.41 ± 20.94 g/L, total RBC count of 1.80 ± 0.68 × 10¹²/L, and total WBC count of 32.47 ± 14.81 × 10⁹/L. Lymphocytes were the predominant WBC type, with a mean absolute count of 16.14 ± 7.27 × 10⁹/L, followed by monocytes at 8.52 ± 4.09 × 10⁹/L and neutrophils at 5.73 ± 2.86 × 10⁹/L. Thrombocyte counts ranged from 78 to 175 cells per 100 WBCs, whereas total solids, representing total plasma protein, ranged from 26.00 to 64.00 g/L.

Only one adult male and one juvenile female were captured; therefore, their hematological findings were reported individually rather than included in group-level statistical summaries (Table 2). The adult male had a PCV of 0.17 L/L, whereas the juvenile female had a PCV of 0.35 L/L and an Hb concentration of 105.00 g/L. MCH and MCHC could not be calculated for the adult male because an Hb value was unavailable. In both individuals, lymphocytes were the predominant WBC type, followed by monocytes. Total solids were 46 g/L in the adult male and 36 g/L in the juvenile female.

**Table 2:** Preliminary hematologic values of Scatophagus argus from Nakhon Si Thammarat, Southern Thailand.

**Parameters**	**SI unit**	**Juvenile male ** **(Mean)**	**Juvenile male ** **(Median)**	**SD**	**Min**	**Max**	**Adult male** **(n = 1)**	**Juvenile female** **(n = 1)**
PCV (n = 23)	L/L	0.24	0.25	0.07	0.11	0.40	0.17	0.35
Hb (n = 17)	g/L	74.41	73.00	20.94	39.00	123.00	NA	105
RBC (n = 24)	×10¹²/L	1.80	1.71	0.68	0.70	3.70	1.68	1.69
MCV (n = 23)	fL	152.65	142.86	67.02	26.70	385.71	101.19	207.10
MCH (n = 17)	pg	23.93	22.88	7.59	15.63	49.45	NA	62.13
MCHC (n = 17)	g/dL	29.29	29.20	2.30	24.58	34.40	NA	30
WBC (n = 24)	×10⁹/L	32.47	30.13	14.81	12.50	59.75	37.25	41.25
Neutrophils (n = 24)	×10⁹/L	5.73	4.60	2.86	1.88	12.10	4.84	7.84
Eosinophils (n = 24)	×10⁹/L	2.08	1.63	1.13	0.63	4.40	1.49	3.71
Basophils (n = 24)	×10⁹/L	0.00	0.00	0.00	0.00	0.00	0.00	0.00
Monocytes (n = 24)	×10⁹/L	8.52	8.16	4.09	3.56	17.93	10.43	10.31
Lymphocytes (n = 24)	×10⁹/L	16.14	14.78	7.27	5.96	29.28	20.49	19.39
Neutrophils (n = 24)	%	17.58	18.00	2.54	13.00	22.00	13	19
Eosinophils (n = 24)	%	6.38	6.00	1.56	4.00	9.00	4	9
Basophils (n = 24)	%	0.00	0.00	0.00	0.00	0.00	0.00	0.00
Monocytes (n = 24)	%	26.25	26.50	3.40	20.00	32.00	28	25
Lymphocytes (n = 24)	%	48.79	50.00	3.68	44.00	57.00	55	47
Thrombocytes (n = 24)	/100 WBCs	107.13	100.00	22.91	78.00	175.00	86	132
Total solids (n = 24)	g/L	41.48	42.00	10.60	26.00	64.00	46	36

NA = Data not available; Hb = Hemoglobin; MCH = Mean corpuscular hemoglobin; MCHC = Mean corpuscular hemoglobin concentration; MCV = Mean corpuscular volume; PCV = Packed cell volume; RBC = Red blood cells; SD = Standard deviation; WBC = White blood cells.

## DISCUSSION

### Blood cell morphology and comparison with other teleosts

The peripheral blood cells of *S. argus* comprised RBCs, WBCs, and thrombocytes, consistent with the cellular composition reported in other teleost species [36, 39]. Tavares-Dias *et al.* [40] described an additional WBC type, termed special granulocytic cells, in Brazilian teleost fishes; however, this cell type was not observed in *S. argus* in the present study. The RBC morphology identified in this study was similar to that previously reported in *S. argus* from Vietnam [41].

Neutrophils in *S. argus* contained abundant pale gray to blue-gray cytoplasm. This appearance differed from that described in sablefish (*Anoplopoma fimbria*) and koi (*Cyprinus carpio*), in which neutrophils exhibited more eosinophilic cytoplasm [36, 42]. Their morphology also differed from that reported in Japanese flathead (*Inegocia japonica*), whose neutrophils displayed bright blue cytoplasm [43]. An important finding of the present study was the close morphological resemblance between neutrophils and monocytes. Nevertheless, monocytes could generally be differentiated by their more deeply basophilic cytoplasm.

Eosinophils in *S. argus* were generally small, round cells containing small, bright eosinophilic granules that resembled those reported in sablefish [42]. The granules had indistinct margins, and small rod-shaped structures were evident in only some cells. Ultrastructural examination using transmission electron microscopy (TEM) is warranted to confirm these morphological characteristics. TEM may also facilitate the identification of other WBC types that are difficult to distinguish using routine light microscopy. In addition, the cytoplasm of some eosinophils appeared basophilic, similar to the eosinophilic cell morphology reported in koi [36].

### Functional significance of blood cell types

This study provides the first detailed morphological and quantitative characterization of the peripheral blood cells of *S. argus*. Although the functions of individual blood cell types were not investigated directly, previous studies have indicated that RBCs are primarily responsible for respiratory gas transport, thrombocytes participate in both coagulation and phagocytosis, and WBCs perform essential immune functions [44–46]. Cytochemical staining should therefore be applied in future studies to characterize the enzymatic properties of cytoplasmic granules and improve understanding of the immune functions of circulating blood cells in *S. argus*. Similar approaches have been used successfully in cururu stingray (*Potamotrygon wallacei*), discus ray (*Paratrygon* *aiereba*), ocellate river stingray (*Potamotrygon motoro*), and Nile tilapia (*Oreochromis niloticus*) [47, 48].

Lymphocytes accounted for 44%–57% of circulating WBCs and were the predominant leukocyte type in *S. argus*. This finding was comparable to the proportions reported in sablefish (66%–96%) [42] and Nile tilapia (80.2%) [46]. Lymphocytes are the principal effector cells of adaptive immunity and can be classified into T and B lymphocytes [49], whereas neutrophils serve fundamental roles in innate immune defense [50]. The predominance of lymphocytes may suggest a substantial contribution of adaptive immune mechanisms to circulating leukocyte responses in *S. argus*. However, this interpretation should be approached with caution because leukocyte proportions alone cannot establish the relative functional importance of innate and adaptive immunity.

Lieschke and Trede [49] reported that T and B lymphocytes develop in distinct hematopoietic organs in fish. The present study examined only peripheral blood cells and did not evaluate hematopoietic tissues. To our knowledge, among the hematopoietic and lymphoid organs of *S. argus*, only the spleen has been examined histologically [51]. Therefore, the tissue origins, maturation pathways, and specific subsets of circulating lymphocytes in this species remain unclear. Further histological, ultrastructural, immunohistochemical, and cytochemical investigations are needed to improve blood cell classification and advance understanding of the immune system of *S. argus*.

### Preliminary hematological values and RIs

According to the American Society for Veterinary Clinical Pathology guidelines [52], datasets comprising 20 or fewer animals may be used to estimate RIs using robust statistical methods, with the lower and upper limits reported together with 90% confidence intervals [53]. Although the sample of juvenile male *S. argus* included 24 individuals and therefore met the minimum numerical requirement for robust RI estimation, the RIs calculated using the referenceIntervals package in R were considered insufficiently reliable for meaningful interpretation. This limitation was likely related to the small sample size, wide dispersion of several variables, and presence of potential outliers. Consequently, the findings were reported as preliminary hematological values rather than formal RIs.

Establishment of species- and population-specific RIs is essential for accurate interpretation of hematological findings in local diagnostic laboratories. Hematological values in fish are influenced by species, age, sex, nutritional status, temperature, salinity, and water quality [21–25]. Future studies should therefore recruit substantially larger and more balanced populations of *S. argus*, including both sexes, multiple age groups, and fish from freshwater, brackish, and marine habitats. Standardized sampling, handling, anticoagulation, analytical procedures, and environmental measurements will also be necessary to establish robust RIs suitable for clinical and research applications.

### Diagnostic and environmental implications

Fish remain in continuous contact with their aquatic environment and are therefore highly responsive to changes in water quality and habitat conditions. Environmental alterations can influence blood cell counts, morphology, and distribution [54]. Hematological assessment is consequently useful for screening fish health, identifying physiological disturbances, and monitoring changes in aquatic ecosystems.

The blood cell descriptions and preliminary hematological values generated in this study provide baseline information that may strengthen the capacity of veterinary diagnostic laboratories and clinical services involved in the health of ornamental, cultured, and wild fish. These data may also support the interpretation of future studies investigating disease, nutrition, husbandry stress, pollutant exposure, and environmental degradation in *S. argus*. Because the species inhabits freshwater, brackish, marine, and mangrove ecosystems, its hematological responses may have potential value as biomarkers of ecosystem health. However, this application will require validation through controlled exposure studies and field investigations that directly relate hematological alterations to defined environmental stressors.

### Strengths and limitations

A major strength of this study was the combined evaluation of blood cell morphology, morphometry, and preliminary quantitative hematological values in wild-caught *S. argus*. The use of blinded assessments by two trained veterinary technicians enhanced the reliability of microscopic cell identification and manual counting. The study also provides an initial foundation for clinical hematology and environmental biomonitoring in a species of increasing aquaculture, ornamental, and ecological importance.

Nevertheless, several limitations should be considered. First, specimens were collected from a single locality; therefore, the findings, particularly the CBC results, should be interpreted cautiously when applied to *S. argus* from other regions or countries. Second, most sampled fish were juvenile males collected from a brackish water environment. The results may therefore not represent females, adults, or populations inhabiting freshwater and marine ecosystems. Third, water quality parameters were unavailable at the sampling site, preventing assessment of the possible effects of temperature, pH, dissolved oxygen, ammonia, nitrite, salinity, and other environmental conditions on the hematological findings. Fourth, the limited sample size prevented the establishment of reliable RIs and limited meaningful comparisons by sex and age. Finally, ultrastructural, cytochemical, immunophenotypic, and functional analyses were not performed, limiting definitive classification of some WBC types and interpretation of their immune functions.

### Future research

Future investigations should include larger, geographically diverse populations of *S. argus* and should balance sampling across sex, age, reproductive stage, and habitat type. Concurrent measurement of water quality, salinity, dietary exposure, season, stress indicators, and disease status would help clarify biological and environmental sources of hematological variation. Formal RIs should be established using standardized preanalytical and analytical procedures. TEM, cytochemical staining, flow cytometry, immunohistochemistry, and molecular approaches should also be considered to characterize leukocyte subtypes and their functions. Longitudinal field studies and controlled pollutant exposure experiments would further determine whether hematological variables in *S. argus* can serve as sensitive and reliable bioindicators of the health of freshwater, brackish, marine, and mangrove ecosystems.

## CONCLUSION

This study provides the first integrated description of peripheral blood cell morphology, morphometry, and preliminary hematological values in wild-caught *S. argus* from Southern Thailand. The circulating blood cells comprised RBCs, WBCs, and thrombocytes. Four WBC types were identified: neutrophils, eosinophils, monocytes, and lymphocytes. Neutrophils and monocytes were the largest leukocytes and showed similar diameters, although monocytes were distinguishable by their more deeply basophilic cytoplasm. Lymphocytes were the predominant WBC type. Juvenile males had a mean PCV of 0.24 ± 0.07 L/L, Hb concentration of 74.41 ± 20.94 g/L, total RBC count of 1.80 ± 0.68 × 10¹²/L, and total WBC count of 32.47 ± 14.81 × 10⁹/L. These findings establish preliminary baseline data for the hematological evaluation of this euryhaline species.

The results have practical value for veterinary diagnostic laboratories, aquaculture health management, ornamental fish medicine, and the assessment of wild fish populations. The morphological descriptions may improve the identification and differentiation of circulating blood cells, particularly neutrophils, monocytes, lymphocytes, and thrombocytes, during routine microscopic examination. The preliminary hematological values may also support future interpretation of physiological responses to disease, nutrition, husbandry practices, environmental stress, and pollutant exposure. Because *S. argus* inhabits freshwater, brackish, marine, and mangrove environments, hematological assessment of this species may have potential application in aquatic ecosystem monitoring.

Nevertheless, the findings should be interpreted as preliminary because most sampled fish were juvenile males collected from a single brackish water locality, and water quality parameters were unavailable. Larger studies involving both sexes, multiple age groups, different habitats, and standardized environmental measurements are required to establish reliable RIs. Further cytochemical, ultrastructural, immunophenotypic, and functional investigations are also needed to confirm leukocyte classification and clarify their immunological roles. Overall, the present study provides an essential foundation for advancing piscine hematology in *S. argus* and supports the future development of this species as a clinically relevant model and potential bioindicator of aquatic environmental health.

## DATA AVAILABILITY

The data generated during the study are included in the manuscript.

## GENERATIVE AI DECLARATION

The authors declare that no generative AI was used in the preparation of this manuscript.

## AUTHORS’ CONTRIBUTIONS

KB, KS, and PP: Conceptualization and methodology. PK, TM, KB, KS, and PP: Investigation and data collection. KB, PK, and TM: Writing–original draft preparation. PK, PP, and TM: Sample collection, data visualization, and data curation. PP: Software and validation. KB, KS, NS, PP, and TNT: Formal data analysis. NS, KS, PP, and TNT: Writing–review and editing. All authors have read and approved the final version of the manuscript.
